# Treatment selection and influencing factors for chronic lymphocytic leukemia: a physician survey in Japan

**DOI:** 10.1007/s10147-024-02645-6

**Published:** 2024-10-21

**Authors:** Junichiro Yuda, Chaochen Wang, Tomoko Terasawa, Masaomi Tajimi, Satoshi Osaga, Moemi Miura, Shori Takaoka, Yoshinori Tanizawa

**Affiliations:** 1 Department of Hematology, National Cancer Center Hospital East, 6-5-1 Kashiwanoha, Kashiwa, Chiba 277-8577 Japan; 2 Japan Drug Development and Medical Affairs, Eli Lilly Japan K.K., 5-1-28 Isogamidori, Chuo-ku, Kobe, Hyogo 651-0086 Japan; 3 Social Survey Research Information Co., Ltd., 10-5 Tomihisacho, Shinjuku-ku, Tokyo, 162-0067 Japan

**Keywords:** Chronic lymphocytic leukemia, Treatment selection, Online survey

## Abstract

**Background:**

Chronic lymphocytic leukemia (CLL) is a rare form of lymphoma in Japan. This study aimed to explore hematologists’ motivations and considerations in making treatment decisions for CLL.

**Methods:**

Responses from hematologists treating CLL, obtained through an online survey, were descriptively analyzed. Subgroup analyses by preferred first-line (1L) treatment, years of clinical experience, and level of interest in CLL were conducted.

**Results:**

Out of 107 hematologists surveyed, 82.2% identified Bruton tyrosine kinase inhibitors (BTKi) as their primary choice for 1L treatment; the reasons included established clinical evidence (61.4%) and oral administration convenience (56.8%). Key factors influencing 1L treatment selection among those favoring BTKi included the presence of 17p deletion, *TP53* mutation, and patient’s fitness status. BTKi was favored by 92.6% of hematologists with < 10 years of clinical experience and by 78.8% with more experience. The main reasons for choosing BTKi included safety (50.0%) and tolerance (46.7%) among hematologists who stated they had a specific interest in CLL and the oral administration route (62.1%) among hematologists with lower interest. When BTKi was used as 1L therapy, venetoclax-based regimens were preferred for second-line treatment. The most common concern about BTKi was substantial out-of-pocket costs.

**Conclusion:**

Although many Japanese hematologists select their treatment based on clinical evidence, variations exist in treatment strategies, possibly associated with hematologists’ experience and interest in CLL. These findings underscore the importance of further promoting evidence-based treatments to ensure that all physicians can make informed decisions. Future research should explore additional factors that influence CLL treatment decisions.

**Supplementary Information:**

The online version contains supplementary material available at 10.1007/s10147-024-02645-6.

## Introduction

Chronic lymphocytic leukemia/small lymphocytic lymphoma (CLL/SLL, henceforth referred to as CLL) arises from the transformation of B lymphocytes into cancerous cells. In the USA, CLL is relatively prevalent among malignant lymphomas, comprising 24.1% of all diagnoses of malignant lymphomas between 2003 and 2008. In contrast, CLL constitutes only 3.2% of malignant lymphomas diagnosed in Japan during the same period [1]. However, the age of CLL onset in Japan does not significantly differ from that observed in other countries [2, 3]. Given CLL’s characteristic slow progression and its higher prevalence in the elderly [4], who may have comorbidities or prone to treatment-related complications, treatment strategies should encompass considerations not only of clinical features and disease status but also patient preferences and the potential impact of treatment on daily life.

The rarity of CLL in Japan may pose challenges for hematologists, limiting the opportunities to treat patients with CLL. Despite recent approvals of new medications such as ibrutinib, acalabrutinib (Bruton tyrosine kinase inhibitors; BTKi), and venetoclax (B-cell lymphoma 2 inhibitor; BCL2i) for clinical use, there exists limited evidential support in Japan for their optimal utilization, including treatment timing, sequencing, and evaluation of treatment efficacy in real-world clinical settings. Although various treatment options are available for CLL, novel therapies may not be considered as a potential choice due to limited evidence or may be overlooked due to lack of experience using them. This could hinder the selection of the best treatment and potentially limit benefits for the patient.

While guidelines offer treatment recommendations, there is limited information on how hematologists make these decisions in Japanese clinical practice. Previous studies have used prescription databases to explore CLL treatment patterns; for instance, Takizawa et al. identified fludarabine alone (17.7%), cyclophosphamide alone (13.7%), and bendamustine/rituximab (8.2%) as commonly prescribed regimens as first-line (1L) treatment [5], while Maruyama et al. noted various treatments such as BTKi (47.7%), bendamustine (17.0%), and venetoclax (13.1%) following discontinuation of initial BTKi [2]. However, these studies did not provide insights into the rationale behind these prescriptions from the perspective of practicing hematologists.

Understanding the decision-making processes of hematologists regarding CLL treatment, including the perception of conventional and novel therapies and the extent of satisfaction and dissatisfaction with available therapies, is crucial to clarifying the potential evidence gap and promoting informed treatment decisions. Therefore, we conducted a survey among hematologists in Japan to assess the current landscape of CLL treatment selection and the factors influencing their treatment choice for CLL. This study aimed to elucidate whether CLL treatment practices in Japan align with current guidelines and detail the decision-making processes employed by hematologists.

## Patients and Methods

### Participants

In this cross-sectional study, an online survey was employed to collect data from hematologists practicing in Japanese hospitals who were treating at least one patient with CLL. Participants were recruited from the registered members of m3.com, a platform dedicated to offering medical information. This research panel is managed by M3, Inc., which has a membership of over 320,000 physicians [6], covering approximately 90% of the registered physicians in Japan [7]. At the time of the survey, 4,475 physicians were registered as hematologists working at hospitals. Given the descriptive nature of the study, a target sample size of 100 was deemed feasible for data collection purposes.

Hematologists meeting the specified criteria and completing the survey underwent scrutiny for inconsistencies in their responses, unusual numerical data, and patterns indicating repeated selection of the same option across questions. Those identified were subsequently excluded from the analysis.

### Survey content

An original questionnaire was developed and refined based on interviews with four hematologists to ensure its validity before the main survey. Notably, these interviewees were excluded from participation in the main survey.

The questionnaire comprised approximately 50 questions, including screening, background, and main questions. In the main part, participants were asked about drug selection and the reasons for 1L and second-line (2L) CLL treatment, points of satisfaction and dissatisfaction about regimens for CLL, access to information, and other aspects of CLL treatment. Regarding drug selection for 1L treatment, participants were asked “For primary treatment of CLL, which treatment do you consider first?” to understand the general trend of treatment selection. Participants indicated their response by choosing from two options: “I will primarily select BTK inhibitors” or “I will primarily select chemotherapy.” Respondents were also asked the extent of agreement with the statement “CLL is the disease of most interest to me in my practice” using a five-point Likert scale. Other major survey questions are provided in Online Resource 1. Participants who completed the survey were remunerated by M3, Inc.

### Statistical analysis

Responses were analyzed using descriptive statistics. Key questions were also described based on subgroups defined by the respondent’s primary treatment choice (BTKis or chemotherapy) for 1L treatment, their years of clinical experience, and their level of interest in CLL. Those who selected “strongly agree” or “agree” on the statement about interest in CLL were classified as “highly interested,” whereas those who selected “do not agree at all,” “do not agree,” or “cannot say either” were categorized as “less interested.”

All statistical analyses were conducted using BellCurve for Excel ver.4.04 and BellCurve Hideyoshi Dplus ver.1.12 (both by Social Survey Research Information in Tokyo, Japan).

### Ethical considerations

Approval for this study was granted by the Ethical Review Board of Medical Corporation TOUKEIKAI Kitamachi Clinic (approval number BGQ09593). This study adhered to the principles outlined in the Declaration of Helsinki and the Ethical Guidelines for Medical and Biological Research Involving Human Subjects in Japan. Prior to the commencement of the survey, informed consent was obtained from all participants.

## Results

**Fig. 1:**
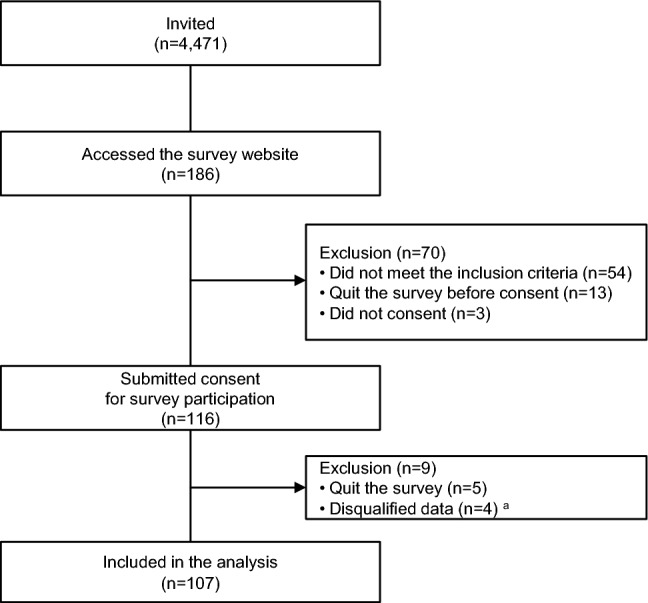
Study population. ^a^Respondents with suspected inappropriate responses (repeatedly choosing the same option for all multiple-choice questions) were excluded

Of the 4,475 registered hematologists, 4 were excluded based on their participation in the interview, and 4,471 were invited to the online survey. Of the 186 physicians (4.2% of those invited) who accessed the survey, 116 consented to participate. After excluding inappropriate responses, 107 respondents were included in the analysis (Fig. 1).

The demographics of the respondents are outlined in Table 1. Hematologists employed at facilities with ≥ 500 beds constituted 59.8% of the total sample. Additionally, 76.6% were certified specialists by the Japanese Society of Hematology, and 74.8% possessed at least 10 years of clinical experience. Thirty-seven physicians (34.6%) were categorized as highly interested in CLL.

**Table 1:** Characteristics of participating hematologists (n = 107)

Age, n (%)	
20–29	4 (3.7)
30–39	35 (32.7)
40–49	24 (22.4)
50–59	28 (26.2)
60–69	13 (12.2)
70 or older	3 (2.8)
Clinical experience with hematologic diseases, n (%)	
Less than 3 years	2 (1.9)
3 to less than 5 years	3 (2.8)
5 to less than 10 years	22 (20.6)
10 years or longer	80 (74.8)
Board-certified member/attending hematologist, n (%)	
Board-certified member of the Japanese Society of Hematology	82 (76.6)
Attending hematologist of the Japanese Society of Hematology	56 (52.3)
None of the above	13 (12.2)
Number of patients with CLL, median (IQR)	5 (2, 7.5)
Number of patients treated with drug therapy for CLL (including watchful waiting after drug therapy), median (IQR)	2 (1, 3)
Number of experienced cases with each drug (excluding clinical trials), median (IQR)	
Ibrutinib	2 (1, 3)
Acalabrutinib	0 (0, 1)
Venetoclax	0 (0, 1)
Type of facility, n (%)	
Cancer center	6 (5.6)
University hospital	29 (27.1)
Other national/public hospital	37 (34.6)
General hospital (hospitals other than those listed above)	35 (32.7)
Number of beds, n (%)	
0–19 beds	0 (0.0)
20–99 beds	1 (0.9)
100–199 beds	9 (8.4)
200–499 beds	33 (30.8)
500 beds or more	64 (59.8)
Whether the facility is a designated cancer care hospital, n (%)	
Yes	75 (70.1)
No	30 (28.0)
Do not know	2 (1.9)
Level of interest in CLL, n (%) ^a^	
Highly interested	37 (34.6)
Less interested	70 (65.4)

*CLL* chronic lymphocytic leukemia; *IQR* interquartile range

^a^The level of interest was determined based on respondent’s agreement with the statement, “CLL is the disease of most interest to me in my practice.” Respondents who selected “completely agree” or “agree” were categorized as “highly interested,” while those who chose “do not agree at all,” “do not agree,” or “cannot say either” were categorized as “less interested”

**Fig. 2:**
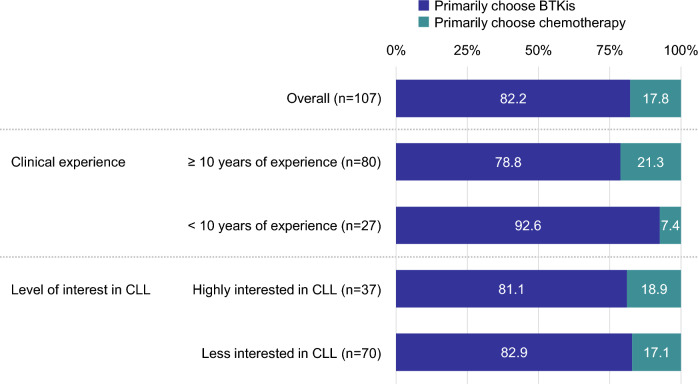
Primary treatment option for first-line CLL treatment. Respondents were asked to select either of two options (BTKis or chemotherapy). BTKis, Bruton tyrosine kinase inhibitors; CLL, chronic lymphocytic leukemia

**Fig. 3:**
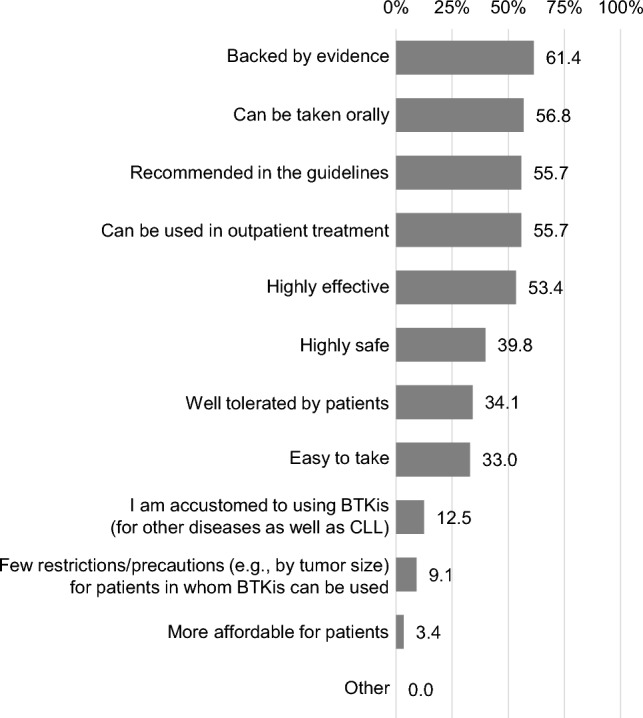
Reasons for choosing BTKis as primary treatment option for CLL first-line treatment (n = 88). Respondents were presented with the list of 12 reasons and asked to select all that apply. BTKis, Bruton tyrosine kinase inhibitors; CLL, chronic lymphocytic leukemia

When presented with two choices, BTKi or chemotherapy, 82.2% (n = 88) of the respondents indicated BTKi as their primary choice for 1L CLL treatment (Fig. 2). The predominant reason for choosing BTKi was “backed by evidence” (61.4%), followed by “can be taken orally” (56.8%), “recommended in the guidelines” (55.7%), and “can be used in outpatient treatment” (55.7%) (Fig. 3). Conversely, among those favoring chemotherapy (n = 19; 17.8%), the leading reason for not selecting BTKi was “the need to continuously take them” (n = 7, 7/19 = 36.8%).

**Fig. 4:**
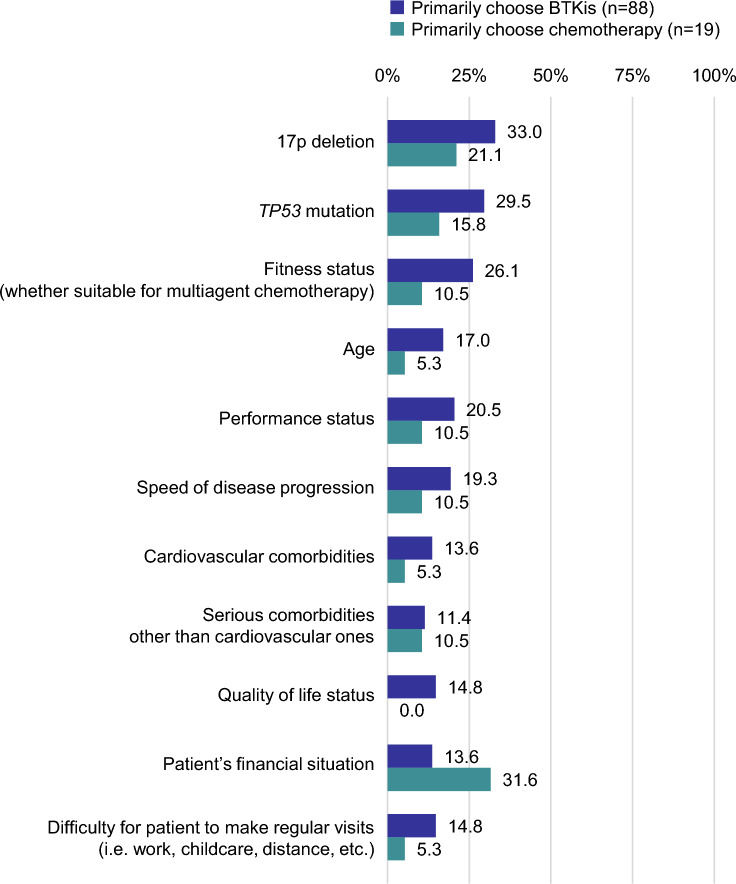
Extent of influence on selection of first-line treatment (BTKis or chemotherapy) according to respondents’ primary choice of treatment. Respondents indicated the extent of influence of each factor on a five-point scale from 1 “No influence at all” to 5 “A great influence.” The percentages of respondents who selected “5” are shown. BTKis, Bruton tyrosine kinase inhibitors

The factors influencing their 1L treatment selection were stratified by primary treatment option. Among the BTKi group, the commonly selected factors that have “a great influence” included 17p deletion (33.0%), *TP53* mutation (29.5%), and patient fitness status (26.1%). In contrast, the chemotherapy group frequently identified the patient’s financial situation (31.6%) as an influencing factor (Fig. 4).

The primary treatment preferences for 1L treatment were further analyzed based on respondents’ clinical experience. When divided into two groups—those with ≥ 10 years of experience (n = 80) and those with < 10 years of experience (n = 27)—78.8% of the more experienced group opted for BTKi therapy, whereas 92.6% of those with less experience made the same choice (Fig. 2).

**Fig. 5:**
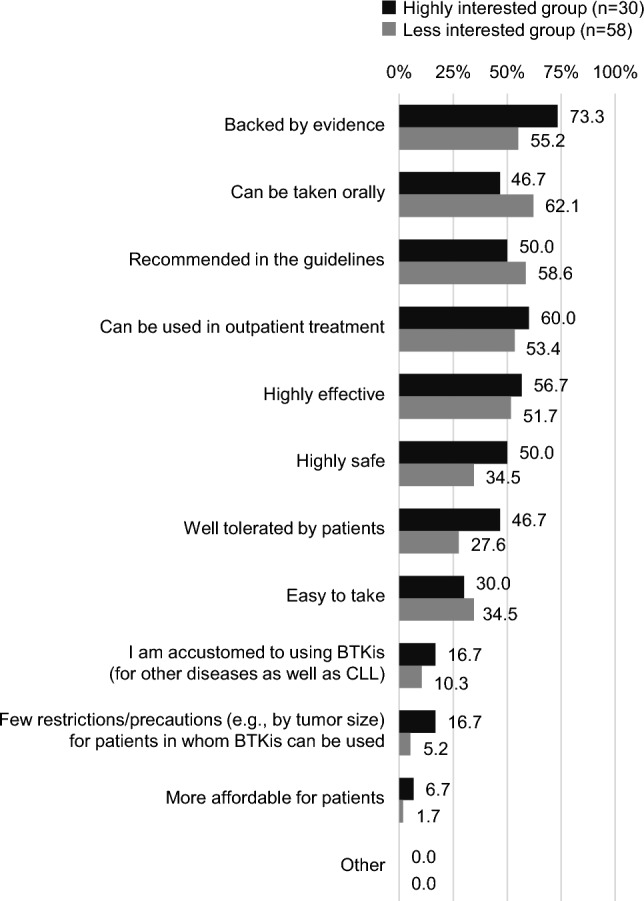
Reasons for choosing BTKis as primary treatment option for first-line CLL treatment based on level of interest in CLL. BTKis, Bruton tyrosine kinase inhibitors; CLL, chronic lymphocytic leukemia

The data were also divided based on respondents’ level of interest in CLL. BTKis were commonly preferred by both groups: 81.1% in the highly interested group and 82.9% in the less interested group (Fig. 2). However, the reasons for choosing BTKis exhibited slight variations. The highly interested group prioritized evidence-based characteristics (73.3%), safety (50.0%), and patient tolerability (46.7%). Conversely, the less interested group favored practical factors such as ease of oral administration (62.1%) and compliance with guideline recommendations (58.6%), while reporting 55.2% for evidence-based characteristics, 34.5% for safety, and 27.6% for patient tolerability (Fig. 5).

**Fig. 6:**
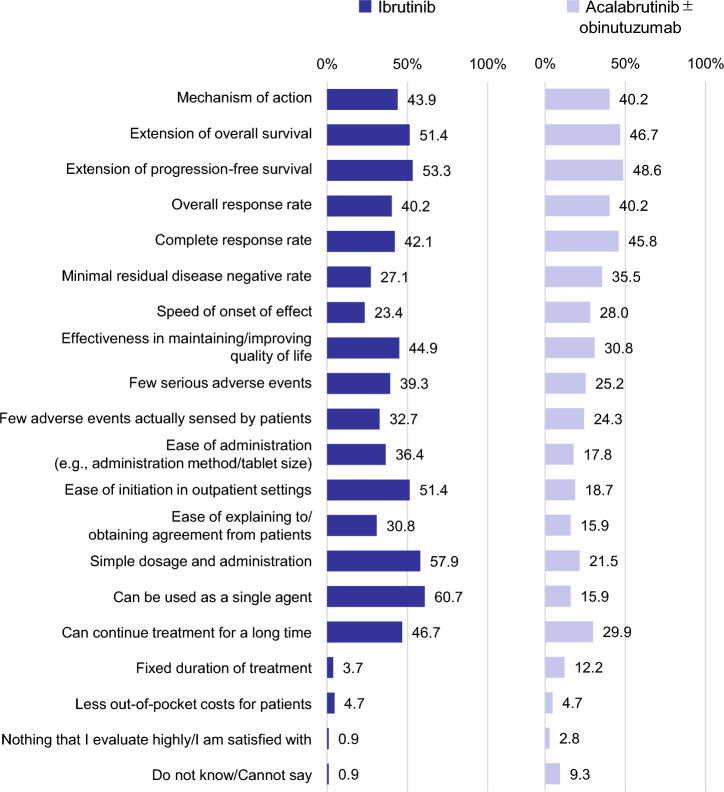
Factors highly evaluated/satisfactory about BTKis for first-line treatment (n = 107). For each regimen used for first-line CLL treatment, respondents were asked what they evaluate highly or are satisfied with and selected all that apply from the 20 factors listed

Regarding the evaluation of BTKi as 1L therapy, nearly half of respondents expressed satisfaction with the extension of progression-free survival (PFS) (53.3% and 48.6%, for ibrutinib and acalabrutinib ± obinutuzumab, respectively), extension of overall survival (OS) (51.4% and 46.7%), and to a slightly lesser extent, attainment of complete response (CR) (42.1% and 45.8%). Additionally, ibrutinib received high percentages of satisfaction for its suitability for monotherapy (60.7%), straightforward dosage and administration (57.9%), ease of initiation in outpatient settings (51.4%), and long-term treatment potential (46.7%) (Fig. 6). Conversely, the points of dissatisfaction regarding BTKi included substantial out-of-pocket costs for patients (42.1% and 43.9%, for ibrutinib and acalabrutinib ± obinutuzumab, respectively) and the absence of fixed treatment duration (36.4% and 23.4%).

Table 2 illustrates the respondents’ primary treatment option as 2L treatment based on the regimen and the reason for discontinuing 1L therapy. In cases where a BTKi was discontinued because of therapy resistance, venetoclax ± rituximab was the most frequently considered 2L therapy (68.2% and 73.8% after ibrutinib- and acalabrutinib-resistance, respectively). If a BTKi was discontinued because of intolerance, some respondents opted for another BTKi as their primary choice of 2L treatment. In the event of ibrutinib intolerance, acalabrutinib ± obinutuzumab and venetoclax ± rituximab were selected as 2L therapies by 46.7% and 43.9% of respondents, respectively. Similarly, following acalabrutinib intolerance, ibrutinib and venetoclax ± rituximab were chosen by 27.1% and 61.7%, respectively. When chemotherapy was the 1L treatment, regardless of the reason for discontinuation, over 70% of respondents favored BTKi as the 2L regimen.

**Table 2:** Primary choice for second-line therapies according to regimens and reasons for discontinuation of first-line treatment (n = 107)

First-line treatment	Reason for discontinuation	Second-line treatment to consider, n (%)			
		Ibrutinib	Acalabrutinib ± obinutuzumab	Venetoclax ± rituximab	Chemotherapy
Ibrutinib	Resistance	5 (4.7)	18 (16.8)	73 (68.2)	11 (10.3)
	Intolerance	2 (1.9)	50 (46.7)	47 (43.9)	8 (7.5)
Acalabrutinib	Resistance	11 (10.3)	3 (2.8)	79 (73.8)	14 (13.1)
	Intolerance	29 (27.1)	2 (1.9)	66 (61.7)	10 (9.3)
Chemotherapy	Refractory (primary resistance)	46 (43.0)	35 (32.7)	22 (20.6)	4 (3.7)
	Recurrence after response (recurrence)	46 (43.0)	30 (28.0)	26 (24.3)	5 (4.7)
	Intolerance	53 (49.5)	30 (28.0)	20 (18.7)	4 (3.7)

When divided based on respondents’ clinical experience, the more experienced group selected venetoclax ± rituximab (48.8%) and acalabrutinib ± obinutuzumab (42.5%) as 2L therapy following ibrutinib intolerance. The other group with less experience favored acalabrutinib ± obinutuzumab (59.3%), followed by venetoclax ± rituximab (29.6%).

**Fig. 7:**
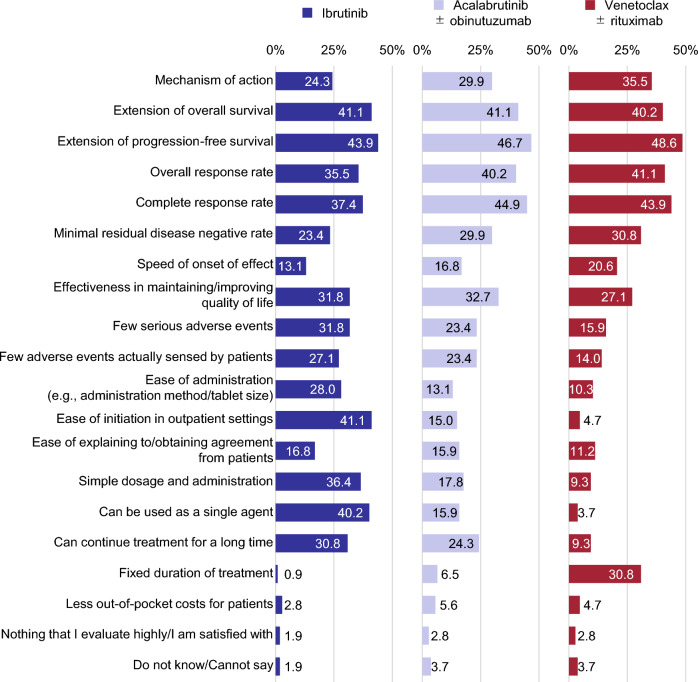
Factors highly evaluated/satisfactory about each regimen used for second-line treatment (n = 107). For each regimen used for second-line CLL treatment, respondents were asked what they evaluate highly or are satisfied with and selected all that apply from the 20 factors listed

Hematologists expressed satisfaction with all three regimens (ibrutinib, acalabrutinib ± obinutuzumab, and venetoclax ± rituximab) for 2L treatment for their PFS extension effect (43.9%, 46.7%, and 48.6%, respectively), OS extension effect (41.1%, 41.1%, and 40.2%), CR rate (37.4%, 44.9%, and 43.9%), and overall response rate (35.5%, 40.2%, and 41.1%) (Fig. 7). Notably, respondents also expressed satisfaction with ibrutinib for its practical benefits, such as ease of initiation in outpatient settings (41.1%), suitability for monotherapy (40.2%), straightforward dosage and administration (36.4%), and potential for long-term use (30.8%). Acalabrutinib ± obinutuzumab was noted for its efficacy in maintaining or improving quality of life (QOL, 32.7%), while venetoclax ± rituximab received positive feedback for its mechanism of action (35.5%) and fixed treatment duration (30.8%).

Moreover, when evaluating the regimens used for 2L treatment, respondents expressed dissatisfaction with substantial out-of-pocket costs for patients (36.4% and 37.4% for ibrutinib and acalabrutinib ± obinutuzumab, respectively) and the absence of a fixed treatment duration (35.5% and 26.2%), echoing concerns raised regarding their use as 1L treatments. For venetoclax ± rituximab, the primary point of discontent was the substantial out-of-pocket cost for patients (34.6%), followed by the inability to initiate treatment in outpatient settings (24.3%).

## Discussion

The current study represents a comprehensive exploration of perspectives and essential factors regarding hematologists’ treatment selection for CLL in Japan. The findings revealed some variability in treatment preferences and rationales among hematologists.

Many hematologists indicated BTKi as their primary choice for 1L treatment, consistent with recommendations in Japanese, American, and European guidelines [3, 4, 8]. The Japanese guidelines for the treatment of CLL largely align with those in the USA and Europe in terms of approved drug classes. Exceptions include the combination therapy of venetoclax + obinutuzumab, which is an approved and preferred regimen for untreated CLL in both the USA [3] and Europe [8]. Another exception is idelalisib + rituximab, which is used for untreated CLL (in Europe) [8] and for relapsed/refractory CLL (in the USA) [3]. Neither regimen is approved in Japan and thus not included in current guidelines.

The number of new drugs available for the treatment of CLL is expected to increase in Japan in the future with potential candidates including the noncovalent BTKi pirtobrutinib [9], phosphatidylinositol 3-kinase inhibitors (idelalisib and duvelisib), and monoclonal antibodies [10]. Therefore, with the changing treatment landscape, hematologists should be aware of the latest treatment options. This can be achieved by increasing awareness and understanding of the latest guidelines to inform treatment selection in the future.

The survey found that the selection of BTKi among physicians with a high interest in CLL was based on safety profile and tolerability of BTKi. Hematologists who predominantly opted for BTKi noted the significance of 17p deletion, *TP53* mutation, and fitness status in their decision-making process regarding 1L therapy. This suggests that hematologists adhere to guidelines recommending molecularly targeted therapy, particularly BTKi, for patients with CLL at high risk in terms of prognosis, such as those with 17p deletion or *TP53* mutation [4]. Furthermore, the consideration of fitness status indicates that chemotherapy may be reserved for patients capable of tolerating its associated adverse effects, which was consistent with our data showing approximately 20% of the respondents reported dissatisfaction with serious adverse events related to chemotherapy. Conversely, hematologists favoring chemotherapy pointed to the patient’s financial situation as a significant determinant in their treatment selection. Notably, 16.8% and 26.2% of all respondents reported satisfaction with the low out-of-pocket costs for bendamustine + rituximab and fludarabine + cyclophosphamide + rituximab, respectively. The results indicated that some hematologists may choose chemotherapy due to its relatively lower out-of-pocket costs.

The study also revealed that treatment choices might differ based on the hematologists’ experience. Over 20% of respondents with ≥ 10 years of clinical experience selected chemotherapy as 1L treatment, deviating somewhat from current guideline recommendations. Interestingly, our data showed the level of agreement with the statement “Guidelines should be the first priority in the treatment of CLL” were comparable between hematologists with ≥ 10 years and < 10 years of experience (51.3% vs. 48.1%). Given the latest guidelines recommendation with BTKi, it is speculated that hematologists with longer experience may base their decisions on past experiences, refer to earlier guideline recommendations, or have more frequent opportunities to treat patients for whom chemotherapy is a priority.

The findings also revealed that hematologists with a high interest in CLL tended to opt for BTKi based on evidence and safety considerations, whereas those less interested in CLL did so in line with guideline recommendations or for the convenience of its oral administration. Hematologists who did not have high interest in CLL may be managing a broad spectrum of hematologic diseases, which could impede their ability to dedicate time to gather information and accumulate clinical experience specifically on CLL. For such hematologists, adhering to guidelines is considered a sound practice when making treatment decisions. However, our results indicated that only 58.6% of those not having high interest in CLL selected guideline recommendations as a rationale for selecting BTKi. Along with consideration of individual characteristics of patients and drugs, understanding of the current guidelines should be promoted so that hematologists can refer to them when facing difficulties in treatment selection. Additionally, there is a need for educational resources such as online courses, videos, and guidelines provided by organizations like the American Society of Hematology [11], offering readily accessible information to assist hematologists in obtaining necessary guidance when required.

Hematologists highly value BTKis as 1L therapy due to their efficacy, effectiveness in maintaining or enhancing QOL, and lower incidence of adverse events. Moreover, BTKis were satisfying hematologists for their practical aspects including ease of initiation in outpatient settings, suitability for standalone use, and simple dosage and administration. Regarding the treatment duration with BTKi, on the one hand, many hematologists expressed satisfaction with its ability to facilitate long-term treatment, which suggests they prefer the treatments that do not significantly impact patients’ daily lives. On the other hand, others expressed dissatisfaction with the absence of fixed treatment duration for BTKis. This contradiction indicated varying preferences regarding long-term treatment approaches. Additionally, hematologists commonly expressed dissatisfaction with the high out-of-pocket costs associated with BTKis, despite Japan’s universal health insurance system. However, treatment with oral medications might offer financial advantages by enabling patients to continue working or minimizing the impact of treatment on their daily lives. Therefore, it is crucial for hematologists to discuss patient concerns and the benefits of OS and PFS extension with BTKis, empowering patients to make well-informed treatment decisions.

In 2L therapy, the treatment options preferred by many hematologists were venetoclax ± rituximab after resistance to 1L BTKis, venetoclax ± rituximab or another BTKi after intolerance, and BTKi after chemotherapy. Our findings indicate that hematologists’ treatment choices generally adhere to guidelines for 2L therapy [4].

The findings also indicated that the selection of 2L therapy might vary depending on hematologists’ clinical experience. Those with more experience selected venetoclax ± rituximab as the most preferred 2L treatment following ibrutinib intolerance, whereas those with < 10 years of experience selected acalabrutinib ± obinutuzumab as the preferred 2L treatment. Guidelines include acalabrutinib as a well-tolerated option suitable for 2L therapy in patients indicated for BTKis but unable to continue ibrutinib because of adverse events [4]. A comparative trial of acalabrutinib vs. ibrutinib in CLL demonstrated lower discontinuation rates due to adverse events with acalabrutinib [12]. The inclination to using different BTKi after first BTKi may stem from a better understanding of the efficacy and safety profiles of each BTKi. However, it is noteworthy that some respondents expressed a preference for ibrutinib after acalabrutinib intolerance, indicating a potential lack of familiarity with the distinct safety profiles of these medications.

BTKis were highly preferred as 2L therapy, particularly emphasizing their efficacy. Conversely, one of the primary rationales for selecting a BCL2i as 2L therapy was the belief that a drug with a different mechanism of action would be more advantageous. For BTKis, hematologists commonly identified similar dissatisfaction as in 1L, whereas for venetoclax ± rituximab, the most common concerns were high out-of-pocket costs and the inability to initiate treatment in an outpatient setting. Given the rarity of CLL, hematologists may have limited exposure to decision-making regarding drug therapy for CLL, especially in selecting 2L therapy due to the condition’s slow progression. Our findings indicate that convenience-related issues, such as costs, treatment duration, and hospitalization requirements, might pose obstacles to the adoption of new drugs despite the recognition of their efficacy. Thus, it is imperative for hematologists to provide patients with information grounded in a comprehensive understanding of available evidence and engage in collaborative discussions.

Using the research panel of m3.com, this study managed to capture the perspectives of a diverse range of hematologists, including not only CLL experts but also hematologists with varying clinical backgrounds and levels of interest in CLL management. Although previous studies have shown the realities of drug prescription, the reasons and intentions behind treatment decisions cannot be understood by the prescription database. With the use of online surveys, it is possible to directly assess the attitudes and motivations of hematologists. The findings offered a current insight into treatment strategies within real-world clinical settings, shedding light on the motivations and considerations driving hematologists’ treatment decisions.

Nevertheless, the current study has some limitations worth noting. First, background details were self-disclosed by participants via an online survey. Second, due to the descriptive nature of the study, we did not conduct statistical testing to look into the potential variations in attitudes associated with respondents’ backgrounds, and no causal relationships could be evaluated. Nonetheless, we employed descriptive statistics to explore these aspects, yielding valuable and clinically relevant insights. Finally, the absence of a well-established testing framework for measurable residual disease and the somatic hypermutation status of the immunoglobulin heavy chain variable region, recognized as a prognostic factor [4], could impact CLL treatment decisions. However, these aspects were not addressed in the current survey. Moreover, these tests are not presently covered by insurance and are scarcely used in Japanese clinical practice. Subsequent research endeavors should tackle these issues, as they may contribute to treatment disparities between facilities equipped to conduct these tests and those that are not.

In summary, while hematologists in Japan generally make their treatment decisions for CLL based on available evidence, some make subjective judgments that differ from those recommended by current guidelines. The variety of approaches to treatment selection indicates that discussions with and learning from other hematologists with different clinical background and the use of readily accessible educational resources may lead to an improved decision-making process. Additionally, the varied treatment approaches among hematologists highlight the importance of shared decision-making with patients to thoroughly consider potential treatment options. Ensuring that all hematologists have access to, comprehend, and use the latest evidence is crucial to offering optimal treatment options for patients.

## Supplementary Information

Below is the link to the electronic supplementary material.

Supplementary file 1 (PDF 318 KB)

## Data Availability

The data that support the findings of this study are not openly available due to reasons of sensitivity and are available from the corresponding author upon reasonable request.

## References

[1] Chihara D, Ito H, Matsuda T et al (2014) Differences in incidence and trends of haematological malignancies in Japan and the United States. Br J Haematol 164:536–545 24245986 10.1111/bjh.12659 PMC3907701

[2] Maruyama D, Wang C, Tanizawa Y et al (2023) Treatment patterns in patients with chronic lymphocytic leukemia/small lymphocytic lymphoma post covalent Bruton tyrosine kinase inhibitor treatment: a Japanese claims database study. J Clin Exp Hematop 63:219–229 38148012 10.3960/jslrt.23032 PMC10861376

[3] Wierda WG, Brown J, Abramson JS, et al (2024) NCCN Clinical Practice Guidelines in Oncology (NCCN Guidelines) Chronic Lymphocytic Leukemia/Small Lymphocytic Lymphoma Version 1.2024. In: Treatment by Cancer Type. Available via https://www.nccn.org/guidelines/category_1. Accessed Mar 2024

[4] Japanese Society of Hematology (2023) Guidelines for the Practice of Hematopoietic Tumors, 2023. Tokyo

[5] Takizawa J, Izutsu K, Nagai H et al (2021) Real world treatment practices for chronic lymphocytic leukemia in Japan: an observational database research study (CLIMBER-DBR). J Clin Exp Hematop 61:126–134 34092721 10.3960/jslrt.20044 PMC8519248

[6] M3, Inc. (2024) Research Panel Provision Service. In: M3, Inc. website. Available via https://corporate.m3.com/service/panel-provide/. Accessed Apr 2024

[7] Ministry of Health, Labour and Welfare (2022) Overview of Statistics of Physicians, Dentists and Pharmacists 2020. In: Ministry of Health, Labour and Welfare website. Available via https://www.mhlw.go.jp/toukei/saikin/hw/ishi/20/dl/R02_gaikyo-b1.pdf. Accessed Apr 2024

[8] Eichhorst B, Robak T, Montserrat E et al (2021) Chronic lymphocytic leukaemia: ESMO clinical practice guidelines for diagnosis, treatment and follow-up. Ann Oncol 32:23–33 33091559 10.1016/j.annonc.2020.09.019

[9] Cool A, Nong T, Montoya S et al (2024) BTK inhibitors: past, present, and future. Trends Pharmacol Sci 45:691–707 39025681 10.1016/j.tips.2024.06.006 PMC11864106

[10] Islam P (2023) Current treatment options in relapsed and refractory chronic lymphocytic leukemia/small lymphocytic lymphoma: a review. Curr Treat Options Oncol 24:1259–1273 37407887 10.1007/s11864-023-01112-0

[11] American Society of Hematology (2024) Education - Hematology.org. In: American Society of Hematology website. Available via https://www.hematology.org/education. Accessed Apr 2024

[12] Byrd JC, Hillmen P, Ghia P et al (2021) Acalabrutinib versus ibrutinib in previously treated chronic lymphocytic leukemia: results of the first randomized phase III trial. J Clin Oncol 39:3441–3452 34310172 10.1200/JCO.21.01210 PMC8547923

