# Supplementary material for: Treatment selection and influencing factors for chronic lymphocytic leukemia: a physician survey in Japan
Source: Int J Clin Oncol. 2024 Oct 21;30(1):157–67. doi: 10.1007/s10147-024-02645-6 (PMC11700057; doi:10.1007/s10147-024-02645-6)
Supplement: Supplementary file 1 — (PDF 318 KB) [file 10147_2024_2645_MOESM1_ESM.pdf]

International Journal of Clinical Oncology

Online Resource

Treatment selection and influencing factors for chronic lymphocytic leukemia: a physician survey in Japan

Junichiro Yuda[1], Chaochen Wang[2], Tomoko Terasawa[2], Masaomi Tajimi[2], Satoshi Osaga[2], Moemi Miura[3], Shori Takaoka[3], Yoshinori Tanizawa[2]

[1]Department of Hematology, National Cancer Center Hospital East

6-5-1 Kashiwanoha, Kashiwa, Chiba, 277-8577 Japan

[2]Japan Drug Development and Medical Affairs, Eli Lilly Japan K.K.

5-1-28 Isogamidori, Chuo-ku, Kobe, Hyogo, 651-0086 Japan

[3]Social Survey Research Information Co., Ltd.

10-5 Tomihisacho, Shinjuku-ku, Tokyo, 162-0067 Japan

Correspondence

Chaochen Wang, PhD

Japan Value, Evidence, and Outcomes (VEO), Japan Drug Development and Medical Affairs, Eli Lilly Japan K.K.

Address: 5-1-28 Isogamidori, Chuo-ku, Kobe, Hyogo, 651-0086, Japan.

Fax: +81-78-242-9526

Telephone: +81-78-242-4389

Email: wang_chaochen@lilly.com

Online Resource 1 Questionnaire

1.

We would like to ask you about selection between BTK[a] inhibitors and chemotherapy for first-line treatment. For primary treatment of CLL,[b] which treatment do you consider first? (Please check one.)

___ I will primarily select BTK inhibitors

___ I will primarily select chemotherapy

[a] Bruton’s tyrosine kinase

[b] Chronic lymphocytic leukemia

2. (If “I will primarily select BTK inhibitors” is checked)

Please tell us the reasons why you would consider BTK inhibitors as the primary treatment option for first-line CLL treatment. (Please select all that apply.)

___ They are recommended in the guidelines

___ I am accustomed to using them (for other diseases as well as CLL)

___ They are backed by evidence

___ They are well tolerated by patients

___ They are highly effective

___ They are very safe

___ There are few restrictions/precautions (e.g., by tumor size) for the patients in which they can be used

___ They can be used in outpatient treatment

___ They are an oral drug

___ They are easy to take

___ They are more affordable for patients

___ Other

3. (If "I will primarily select BTK inhibitors" is not checked)

Please tell us the reasons behind not consider BTK inhibitors as the primary treatment option for first-line CLL treatment. (Please select all that apply.)

___ They have not been adopted at my facility

___ I have no experience using them

___ There is still little evidence

___ They are not very effective

___ Safety concerns

___ Restrictions/precautions (e.g., by tumor size) for the patients in which they can be used

___ The need for patients to continue taking them

___ They are difficult to take

___ They are very expensive for patients

___ Chemotherapy is more effective

___ I have more experience with chemotherapy

___ Other

4.

We would like to ask you about selection between BTK inhibitors and chemotherapy for first-line CLL treatment. Please tell us the degree of impact each of the factors below has on selecting between BTK inhibitors and chemotherapy. (Please check one per row.)

	On your treatment selection between BTK inhibitors and chemotherapy for first-line treatment				
	No influence at all	←	Cannot say either	→	A great influence
17p deletion	□	□	□	□	□
TP53 mutation	□	□	□	□	□
Fit/Unfit (whether indicated for multi-agent chemotherapy)	□	□	□	□	□
Age	□	□	□	□	□
PS[a]	□	□	□	□	□
Speed of disease progression	□	□	□	□	□
Cardiovascular comorbidities	□	□	□	□	□
Serious comorbidities other than cardiovascular ones	□	□	□	□	□
QOL[b] status	□	□	□	□	□
Patient’s financial situation	□	□	□	□	□
Difficulty for patient to make regular visits (i.e., work, childcare, distance, etc.)	□	□	□	□	□

[a] Performance status

[b] Quality of life

5.

Please tell us what you evaluate highly or are satisfied with for the regimens used for first-line CLL treatment. (Please select all that apply for each column.)

	Ibrutinib	Acalabrutinib ± obinutuzumab	BR (bendamustine + rituximab)	FCR (fludarabine + cyclophosphamide + rituximab)
Mechanism of action	□	□	□	□
OS[a] extension effect	□	□	□	□
PFS[b] extension effect	□	□	□	□
Overall response rate (ORR)	□	□	□	□
Complete response (CR) rate	□	□	□	□
Minimal residual disease (MRD) negative rate	□	□	□	□
Speed of onset of effect	□	□	□	□
Effectiveness in maintaining/improving QOL	□	□	□	□
Few serious adverse events	□	□	□	□
Few adverse events actually sensed by patients	□	□	□	□
How easy it is to take (e.g., administration method/tablet size)	□	□	□	□
How easy it is to introduce on an outpatient basis	□	□	□	□
How easy it is to explain to/obtain agreement from patients	□	□	□	□
Simple dosage and administration	□	□	□	□
Can be used as a single agent	□	□	□	□

	Ibrutinib	Acalabrutinib ± obinutuzumab	BR (bendamustine + rituximab)	FCR (fludarabine + cyclophosphamide + rituximab)
Can continue treatment for a long time	□	□	□	□
Fixed duration of treatment	□	□	□	□
Lower out-of-pocket cost for patients	□	□	□	□
There are no factors that I evaluate highly/am satisfied with	□	□	□	□

Do not know/Cannot say	□	□	□	□

[a] Overall survival

[b] Progression-free survival

6.

Please tell us what you are dissatisfied with or would like to see improved for the regimens used for first-line CLL treatment. (Please select all that apply for each column.)

	Ibrutinib	Acalabrutinib ± obinutuzumab	BR (bendamustine + rituximab)	FCR (fludarabine + cyclophosphamide + rituximab)
Mechanism of action	□	□	□	□
OS extension effect	□	□	□	□
PFS extension effect	□	□	□	□
Overall response rate (ORR)	□	□	□	□
Complete response (CR) rate	□	□	□	□
Minimal residual disease (MRD) negative rate	□	□	□	□
Speed of onset of effect	□	□	□	□
Effectiveness in maintaining/improving QOL	□	□	□	□
Many serious adverse events	□	□	□	□
Many adverse events actually sensed by patients	□	□	□	□
How difficult it is to take (e.g., administration method/tablet size)	□	□	□	□
Cannot be introduced on an outpatient basis	□	□	□	□
How difficult it is to explain to/obtain agreement from patients	□	□	□	□
Dosage and administration are not simple	□	□	□	□

	Ibrutinib	Acalabrutinib ± obinutuzumab	BR (bendamustine + rituximab)	FCR (fludarabine + cyclophosphamide + rituximab)
Cannot be used as a single agent	□	□	□	□
Cannot continue treatment for a long time	□	□	□	□
Duration of treatment is not fixed	□	□	□	□
High out-of-pocket cost for patients	□	□	□	□
There are no factors that I am dissatisfied with/want to see improved	□	□	□	□

Do not know/Cannot say	□	□	□	□

7.

We would like to ask about treatment selection for second-line treatment. In the secondary treatment of CLL, which treatment do you consider first? Please tell us for each first-line treatment.

<First-line treatment>

(Select one for each column)	Ibrutinib-resistance ↓	Acalabrutinib-resistance ↓	Ibrutinib -intolerance ↓	Acalabrutinib -intolerance ↓	Refractory with chemotherapy (primary resistance) ↓	Recurrence after response to chemotherapy (recurrence) ↓	Chemotherapy-intolerance ↓
I will primarily select a BTK inhibitor (ibrutinib)	□	□	□	□	□	□	□
I will primarily select a BTK inhibitor (acalabrutinib ± obinutuzumab)	□	□	□	□	□	□	□
I will primarily select BCL2 inhibitors (venetoclax ± rituximab)	□	□	□	□	□	□	□
I will primarily select chemotherapy	□	□	□	□	□	□	□

8.

Please tell us what you evaluate highly or are satisfied with for the regimens used for second-line CLL treatment. (Please select all that apply for each column.)

	Ibrutinib	Acalabrutinib ± obinutuzumab	Venetoclax ± rituximab	BR (bendamustine + rituximab)	FCR (fludarabine + cyclophosphamide + rituximab)
Mechanism of action	□	□	□	□	□
OS extension effect	□	□	□	□	□
PFS extension effect	□	□	□	□	□
Overall response rate (ORR)	□	□	□	□	□
Complete response (CR) rate	□	□	□	□	□
Minimal residual disease (MRD) negative rate	□	□	□	□	□
Speed of onset of effect	□	□	□	□	□
Effectiveness in maintaining/improving QOL	□	□	□	□	□
Few serious adverse events	□	□	□	□	□
Few adverse events actually sensed by patients	□	□	□	□	□
How easy it is to take (e.g., administration method/tablet size)	□	□	□	□	□
How easy it is to introduce on an outpatient basis	□	□	□	□	□
How easy it is to explain to/obtain agreement from patients	□	□	□	□	□
Simple dosage and administration	□	□	□	□	□
Can be used as a single agent	□	□	□	□	□

	Ibrutinib	Acalabrutinib ± obinutuzumab	Venetoclax ± rituximab	BR (bendamustine + rituximab)	FCR (fludarabine + cyclophosphamide + rituximab)
Can continue treatment for a long time	□	□	□	□	□
Fixed duration of treatment	□	□	□	□	□
Lower out-of-pocket cost for patients	□	□	□	□	□
There are no factors that I evaluate highly/am satisfied with	□	□	□	□	□
Do not know/Cannot say	□	□	□	□	□

9.

Please tell us what you are dissatisfied with or would like to see improved for the regimens used for second-line CLL treatment. (Please select all that apply for each column.)

	Ibrutinib	Acalabrutinib ± obinutuzumab	Venetoclax ± rituximab	BR (bendamustine + rituximab)	FCR (fludarabine + cyclophosphamide + rituximab)
Mechanism of action	□	□	□	□	□
OS extension effect	□	□	□	□	□
PFS extension effect	□	□	□	□	□
Overall response rate (ORR)	□	□	□	□	□
Complete response (CR) rate	□	□	□	□	□
Minimal residual disease (MRD) negative rate	□	□	□	□	□
Speed of onset of effect	□	□	□	□	□
Effectiveness in maintaining/improving QOL	□	□	□	□	□
Many serious adverse events	□	□	□	□	□
Many adverse events actually sensed by patients	□	□	□	□	□
How difficult it is to take (e.g., administration method/tablet size)	□	□	□	□	□
Cannot be introduced on an outpatient basis	□	□	□	□	□
How difficult it is to explain to/obtain agreement from patients	□	□	□	□	□
Dosage and administration are not simple	□	□	□	□	□

	Ibrutinib	Acalabrutinib ± obinutuzumab	Venetoclax ± rituximab	BR (bendamustine + rituximab)	FCR (fludarabine + cyclophosphamide + rituximab)
Cannot be used as a single agent	□	□	□	□	□
Cannot continue treatment for a long time	□	□	□	□	□
Duration of treatment is not fixed	□	□	□	□	□
High out-of-pocket cost for patients	□	□	□	□	□
There are no factors that I am dissatisfied with/want to see improved	□	□	□	□	□

Do not know/Cannot say	□	□	□	□	□
